# Diagnostic error in the emergency department: learning from national patient safety incident report analysis

**DOI:** 10.1186/s12873-019-0289-3

**Published:** 2019-12-04

**Authors:** Faris Hussain, Alison Cooper, Andrew Carson-Stevens, Liam Donaldson, Peter Hibbert, Thomas Hughes, Adrian Edwards

**Affiliations:** 1 Cardiff University, Cardiff, UK; 2 London School of Hygiene and Tropical Medicine, London, UK; 3 Macquarie University, Sydney, Australia; 4 John Radcliffe Hospital, Oxford, UK

**Keywords:** Emergency department, Diagnostic error

## Abstract

**Background:**

Diagnostic error occurs more frequently in the emergency department than in regular in-patient hospital care. We sought to characterise the nature of reported diagnostic error in hospital emergency departments in England and Wales from 2013 to 2015 and to identify the priority areas for intervention to reduce their occurrence.

**Methods:**

A cross-sectional mixed-methods design using an exploratory descriptive analysis and thematic analysis of patient safety incident reports. Primary data were extracted from a national database of patient safety incidents. Reports were filtered for emergency department settings, diagnostic error (as classified by the reporter), from 2013 to 2015. These were analysed for the chain of events, contributory factors and harm outcomes.

**Results:**

There were 2288 cases of confirmed diagnostic error: 1973 (86%) delayed and 315 (14%) wrong diagnoses. One in seven incidents were reported to have severe harm or death. Fractures were the most common condition (44%), with cervical-spine and neck of femur the most frequent types. Other common conditions included myocardial infarctions (7%) and intracranial bleeds (6%). Incidents involving both delayed and wrong diagnoses were associated with insufficient assessment, misinterpretation of diagnostic investigations and failure to order investigations. Contributory factors were predominantly human factors, including staff mistakes, healthcare professionals’ inadequate skillset or knowledge and not following protocols.

**Conclusions:**

Systems modifications are needed that provide clinicians with better support in performing patient assessment and investigation interpretation. Interventions to reduce diagnostic error need to be evaluated in the emergency department setting, and could include standardised checklists, structured reporting and technological investigation improvements.

## Background

Diagnostic error occurs more frequently in emergency departments than in the recorded 10–15% of adverse medical events for routine hospital in-patient hospital care [1]. These errors often result in serious patient harm [2, 3], and in the United States of America (USA) these errors are associated with a significant number of deaths per year [4]. However, the reasons for this are not well established. There is growing concern over diagnostic error in United Kingdom (UK) emergency departments given the increase in patient demand in recent years [5–8]. Diagnostic errors have been largely unaddressed across most healthcare settings, including the emergency department [4, 9–11], despite current estimates suggesting one in ten diagnoses are likely to be incorrect [12–14].

Diagnostic error studies are mostly limited to single case sites [15, 16]. Methods include prospective identification of errors by emergency department clinicians [15], retrospective clinical review of closed malpractice claims [17], and review of cases where the diagnosis on admission differs to that on discharge [18]. There is an opportunity to study diagnostic error in patient safety incident reports in parts of the UK as they comprise 0.5% of reports across all settings in the England and Wales national database of over 13 million patient safety incident reports from healthcare organisations [19]. No studies have specifically analysed contributory factors related to diagnostic error from patient safety incident reports in emergency departments [20].

Diagnostic errors are multifactorial in origin [21, 22], involving human and systems related factors [17, 23, 24], and are challenging for healthcare professionals and researchers to address as they involve a range of health conditions [25, 26]. Patient safety incident report analysis can offer a lens onto the causative factors, why errors are happening and what changes can be recommended to reduce the number of diagnostic errors in emergency departments [27]. Studies of primary care patient safety incident reports [19, 28] have been successful in generating practice improvement recommendations [28–30].

The aim of this study was to characterise the nature of reported diagnostic errors in hospital emergency departments in England and Wales from the years 2013 to 2015 and to identify priority areas for intervention to reduce their occurrence.

The objectives were to:

1. Characterise the nature of patient safety incidents related to diagnostic error occurring in emergency departments;
2. Identify common contributory factors that led to diagnostic errors; and
3. Derive recommendations for priority improvement areas in policy and practice.

## Methods

### Study design and setting

This study was a cross-sectional mixed-methods analysis of emergency department patient safety incident reports concerning diagnostic error. Primary data were extracted from the national (England and Wales) database of such incidents, the National Reporting and Learning System (NRLS)**.** A patient safety incident is defined as, “any unintended or unexpected incident that could have harmed or did harm a patient during healthcare delivery” [31]. From 2010, it has been compulsory for all organisations to enter any patient safety incident of high severity. Safety incidents are reported via local risk management systems which contribute batch returns to the NRLS and by Care Quality Commission direct notification [31, 32]. Incidents are usually reported voluntarily by healthcare professionals, mainly doctors and nurses, who were involved with the incident and are done anonymously via an electronic platform (“Datix”), with most incidents being reported by acute trusts. Each patient safety incident report contains structured information about the location of the incident and the reporter’s perception of harm severity. This is complemented by unstructured free-text descriptions of the incident, potential contributory factors and intended actions to prevent reoccurrence. The database has been described in more detail in a study of patient safety-related hospital deaths in England [28, 33].

### Data sampling

**Fig. 1:**
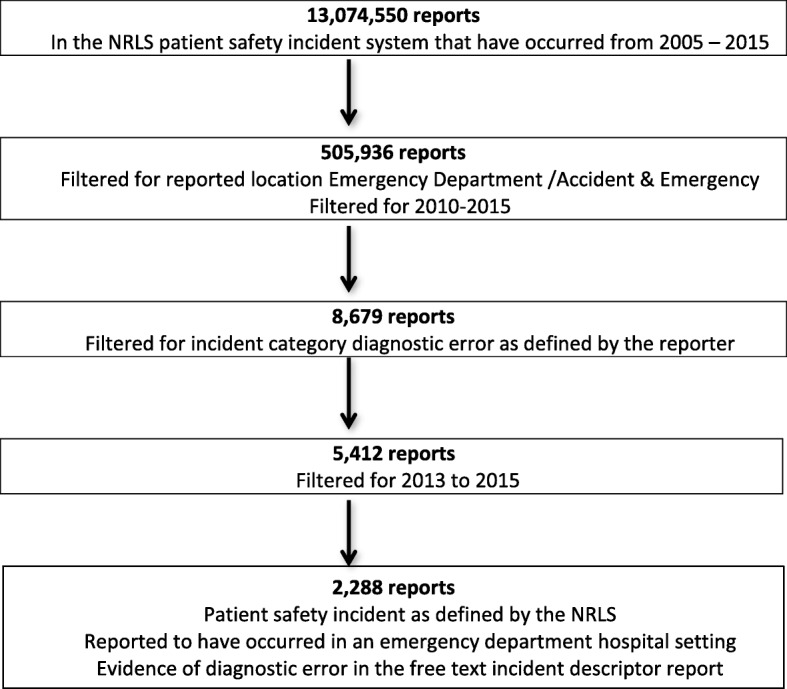
Search strategy and results for NRLS patient safety incident reports describing diagnostic error in emergency department settings 2013–2015

We searched reports in the NRLS for incident category diagnostic error (as defined by the reporter), for emergency department location (as defined within the speciality field), and for reports reported from the years 2013 to 2015, via its electronic database. From 13,074,550 patient safety incident reports within the database we identified 5412 reports (see Fig. 1). From this sample, all reports were read to assess for eligibility criteria. Criteria for including reports in the final analysis were:

1) A patient safety incident as defined by the NRLS had occurred;

2) The patient safety incident occurred in an emergency department hospital care setting outlined in the report;

3) The report did not describe a prevented patient safety incident and.

4) There was evidence in the free text incident descriptor report of a diagnostic error as defined by the Society to Improve Diagnosis in Medicine [4]. These definitions include

- Diagnostic error: “The failure to (a) establish an accurate and timely explanation of the patient’s health problem(s) or (b) communicate that explanation to the patient”
- Wrong diagnosis: “Occurs, for example, if a patient truly having a heart attack is told their pain is from acid indigestion”
- Delayed diagnosis: “Refers to a case where the diagnosis should have been made earlier.”
- Missed diagnosis: “Refers to a patient whose medical complaints are never explained.”

Reports not meeting these criteria were excluded.

### Data coding

We coded each report’s free text according to the classification system developed by Carson-Stevens et al. [33], and used in other studies [29, 30, 34]. This system incorporates coding frameworks different to the NRLS’s own coding framework to record multiple incident types and their contributory factors, outcome and harm severity. For each relevant report, we coded: the type of diagnostic error; the chain of events leading up to the diagnostic error (“contributory incidents”), for example investigation results not followed up or mistakes in interpreting investigations; other “contributory factors”, for example staff fatigue, inadequate staff numbers; and the reported patient outcomes, for example increased level of care, and harm severity. Harm severity classification was based on the World Health Organisation International Classification for Patient Safety definitions [35]. We organised these incidents and factors chronologically through recursive incident analysis [33].

A random sample of 10% of reports was double-coded by AC, with a Cohen’s Kappa score calculated for inter-rater agreement (between FH and AC), and discordance between coders discussed to ensure consistent application of codes and their definitions [36].

### Data analysis

We undertook exploratory descriptive analysis for the frequency of specific diagnoses, the types of diagnostic error and the common incidents and contributory factors occurring.

### Data synthesis

We then conducted thematic analysis, reviewing the constellation of factors and incidents leading to the diagnostic error in relation to their severity of harm [37, 38].

This was done according to the nature of related incidents (e.g. insufficient assessment, imaging reading errors) and associated contributory factors leading to the diagnostic errors. We used the common patterns associated with diagnoses and related incidents to develop a driver diagram, a visual display of what “drives” achievement of an aim, to integrate the most significant themes and their possible interventions [39].

### Results

From 5412 reports which had a diagnostic error defined by the reporter, 2288 (42%) fulfilled our definition of diagnostic error occurring in an emergency department setting and were analysed. From the 10% sample that was double coded, there was a Cohen’s Kappa of 0.868 for inter-rater agreement.

There were 315 (14%) cases of wrong diagnoses and 1973 (86%) cases of delayed diagnoses. No missed diagnoses were described. The three most common conditions involved were fractures, myocardial infarctions and intracranial bleeds, with fractures comprising nearly half of incidents (see Table 1). Of the fractures, hip (22%) and spine (18%) were the most common. The most frequent six diagnoses made up over two-thirds of the incidents. There was sufficient information in 877 reports to assess harm outcomes (38%); of these 176 (20%) documented no harm, 455 (52%) mild harm, 118 (14%) moderate harm, 37 (4%) severe harm and 91 (10%) documented death. The commonest outcomes were: delays in assessment or management, occurring in 1786 reports (78%); repeated visits to or from health care providers (35%); and general deterioration or progression of the condition (12%).

**Table 1:** Frequency of commonly reported diagnoses

Diagnosis associated with diagnostic error	Number of reports	Percentage of total number of reports concerning diagnostic error (%)
Fracture	1007	44
Other/Diagnosis not specified	679	30
Myocardial Infarction	161	7
Intracranial Bleed	140	6
Stroke/CVA	97	4
Acute Abdomen	77	3
Pulmonary Embolism	34	2
Ectopic Pregnancy	31	1
Appendicitis	17	< 1
Ischaemic Limb	15	< 1
DVT	11	< 1
Meningitis	11	< 1
Pneumonia	8	< 1
Total	2288	

Both the wrong and delayed diagnoses had largely common themes for contributory incidents, including: insufficient assessment (32%); inappropriate response to diagnostic imaging/investigations (25%); and failure to order diagnostic imaging/investigations (8%). These three categories of contributory incidents are described in more detail below. In all diagnostic error reports, the most common contributory factors (identified in 1577 reports, 69%) related to staff or human factors: “inadequate skill or knowledge”; “mistake”, “missed task or job to do” (e.g. checking diagnostic test results); and “failure to follow protocol”.

### Insufficient assessment (*n* = 728, 32%)

There were 286/728 (39.%) reports related to fractures, 56 to intracranial bleeds, 39 to acute abdomen cases and 35 to stroke cases. The most common types of fracture in these reports were hip (*n* = 82, 29% of fractures), followed by cervical-spine (*n* = 41, 14% of fractures). Common contributory incidents associated with insufficient assessment included failure to order imaging investigations (*n* = 364), incorrect response to imaging investigations (*n* = 50) and failure to refer patients when indicated (*n* = 36).

### Inappropriate response to diagnostic imaging (*n* = 569, 25%)

These reports included 439/569 (77%) fractures and 19 (3%) intracranial bleeds. The most common fractures were: hip fractures (*n* = 109, 25% of fractures); ankle/foot fractures (*n* = 83, 19% of fractures)); arm fractures (n = 36, 8% of fractures); and hand fractures (*n* = 35, 8% of fractures). Most of these cases had no other reported contributory incidents leading to the diagnostic error (*n* = 434).

### Failure to order diagnostic imaging (*n* = 188, 8%)

Of these reports, 85/188 (45%) related to fractures, 32/188 (17%) to intracranial bleeds and 16/188 (9%) to strokes. The most common fractures included hip (*n* = 23, 27% of fractures) and cervical-spine (*n* = 16, 19% of fractures). Many had no contributory incidents described (*n* = 106), but insufficient assessment was described in 57 reports. Contributory factors included clinician “mistake” (*n* = 32) and “failure to follow protocol” (*n* = 30), with reasons for this including failure to identify indications for imaging from history and examination.

Examples of these reports are presented in Table 2 along with frequencies of contributory factors.

**Table 2:** Contributory factors, outcomes and examples for key contributory incident types

Insufficient assessment reports			
Common Contributory Factors	Common Outcomes	Harm Severity	Example of Report *Delayed diagnosis of intracranial bleed*
• Inadequate skill set/knowledge (*n* = 510, 70%) • Clinician “mistake” (*n* = 192, 26%) • Failure to follow protocol (*n* = 71, 10%)	• Delay in management/assessment (*n* = 659, 91%) • Repeated healthcare visits (*n* = 327, 45%) • General deterioration/progress of condition (*n* = 157, 22%)	353 reports assessed for harm outcome • No harm (*n* = 48, 14%) • Mild harm (*n* = 200, 57%) • Moderate harm (*n* = 52, 15%) • Severe harm (*n* = 16, 4%) • Death (*n* = 37, 10%)	“Patient attended the Emergency department with a head injury. Physician did not undertake neurological observations. Patient reported headache two days post head injury. Lack of assessment by a physician meant that guidelines for head injury were not met and that intracranial bleed was missed.”
Inappropriate response to diagnostic imaging reports			
Common Contributory Factors	Common Outcomes	Harm Outcomes	Example of Report *Delayed diagnosis of cervical-spine fracture*
• Mistake in interpretation of imaging (*n* = 463, 81%) • Inadequate skill set/knowledge (*n* = 352, 62%) • Task to be completed by the clinician (e.g. checking patient notes) (n = 30, 5%).	• Delay in management/assessment occurred (*n* = 476, 84%) • Repeated healthcare visits (*n* = 251, 44%) • General deterioration/progress of condition occurred (n = 55, 10%)	197 reports assessed for harm outcome • No harm (*n* = 55, 28%) • Mild harm (*n* = 104, 53%) • Moderate harm (*n* = 21, 11%) • Severe harm (n = 6, 3%) • Death (*n* = 11, 5%)	“A 75 year-old lady was seen after a fall. She had a Cervical-spine X-ray done which was interpreted as normal. The patient was admitted in Emergency department and discharged the next day. I received a call regarding a missed fracture of C2 (2nd cervical vertebrae). The patient had been admitted at another hospital.”
Failure to order diagnostic imaging reports			
Common Contributory Factors	Common Contributory Factors	Harm Outcomes	Example of Report *Delayed diagnosis of hip fracture*
• Clinician “mistake” (n = 32, 17%) • Failure to follow protocol (n = 30, 16%).	• Delay in management (*n* = 140, 75%) • Repeated visits to/from healthcare providers (*n* = 67, 36%)	62 reports assessed for harm outcomes • No harm (n = 11, 18%) • Mild harm (*n* = 33, 53%) • Moderate harm (n = 7, 11%) • Severe Harm (n = 2, 3%) • Death (*n* = 9, 15%)	“Patient attended emergency department documented on initial assessment that leg shortened and rotated. Patient not x-rayed, sent to Emergency department unit for mobilisation. Unable to mobilise and leg shortened and rotated - X-Ray shows peri-prosthetic hip fracture”

### Severe harm and death reports

From the reports where the harm severity could be determined, 128 resulted in severe harm or death (15%) and were evaluated . Frequent diagnoses included abdominal aortic aneurysm (18 reports), intracranial bleed (15 reports) and pulmonary embolism (8 reports). Related contributory incidents that led to the diagnostic error were similar to the reports overall.

### Potential interventions

**Fig. 2:**
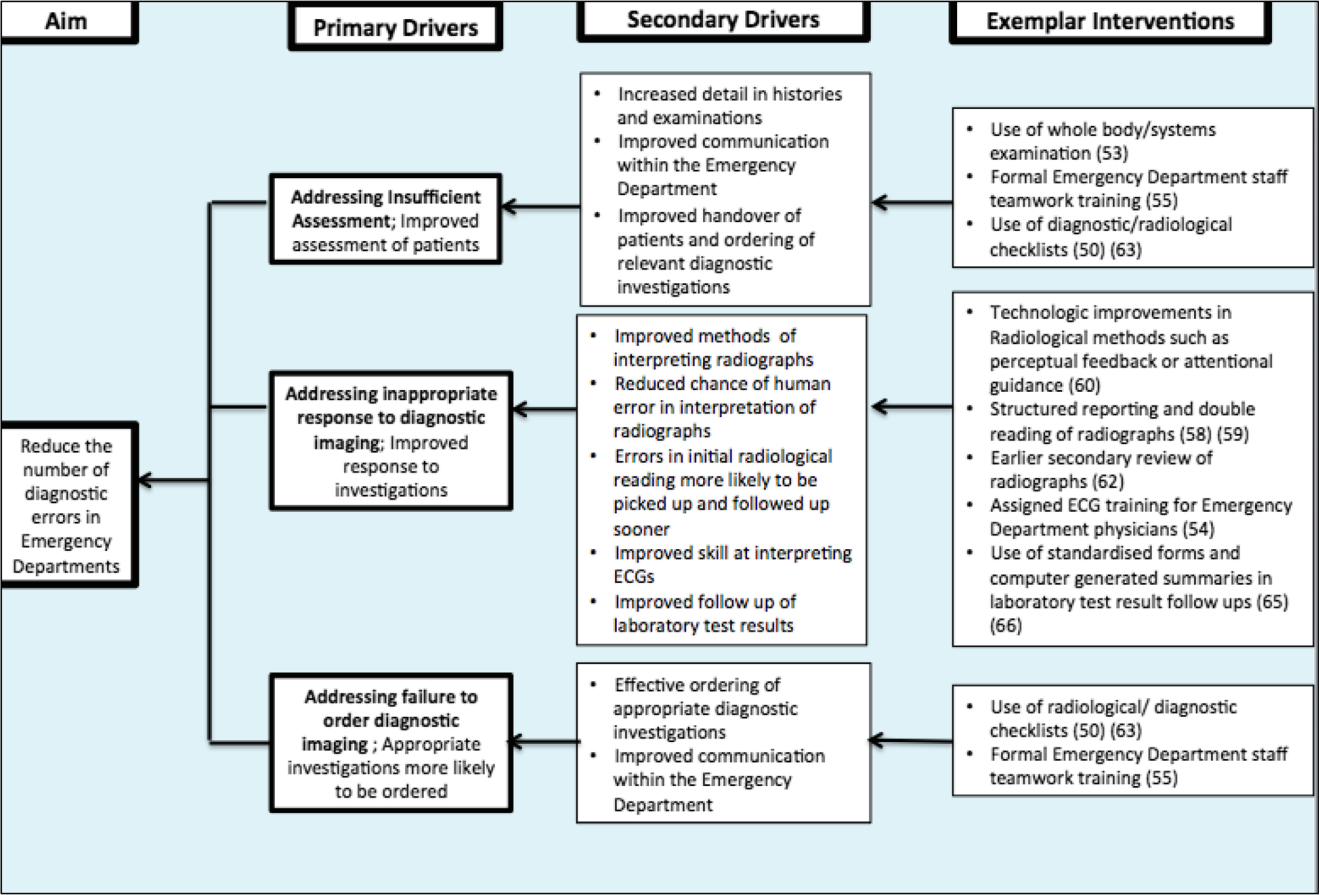
Driver diagram presenting opportunities for reducing diagnostic error in the Emergency Department

Thematic analysis of the reports established that the contributory incidents linked to diagnostic error included insufficient assessment, diagnostic imaging/ investigations interpretation and the ordering and follow up of diagnostic imaging/ investigations. These occurred across a number of diagnoses. Fig. 2 presents a driver diagram of possible interventions [39] that could be examined and evaluated to target these incidents and reduce their occurrence.

## Discussion

### Principal findings

Descriptive and thematic analysis of a large number of nationally reported patient safety incidents of diagnostic error showed that a third related to errors in clinical assessment, a quarter to inappropriate response to diagnostic imaging/investigations and one in 12 to failing to order diagnostic imaging/investigations. Staff human factors, including mistakes, were common. This was consistent for both delayed and wrong diagnoses and across most diagnoses.

Key diagnoses implicated in reports of diagnostic error included hip and cervical spine fractures, myocardial infarctions and intracranial bleeds. Most of these reports detailed incidents of misinterpretation of radiographs, failure to order correct investigations and a lack of sufficient assessment of the patient. Common related contributory factors with these reports concerned inadequate skill and clinician mistakes.

### Strengths and limitations

Underreporting is an established methodological problem in patient safety incident studies but also in similar analyses of data in other high-risk industries [14, 40]. The true incidence of diagnostic error in emergency departments will be higher than we have found. Focussing our analysis on reports where the incident type was ‘diagnostic error’ relies on a reporter suspecting that a diagnostic error has occurred; many diagnostic errors will not be reported as clinicians may be unaware a diagnostic error has occurred. Thus there may be other reports contained within other NRLS categories, such as reports concerning treatment error, which would have not been included in our analysis. No missed diagnoses were coded in the sample. However, for the definition we have used, these may be more likely to present in primary care than the emergency department and such cases may be less likely to be coded as a safety incident by hospital staff [41, 42]. The NRLS is known to have limitations, with incident reporting often influenced by campaigns and alerts that raise awareness of certain incidents and disease, and its reports criticised for having poor data standardisation [43]. Development of the Patient Safety Incident Management System (DPSIMS) is currently in progress to replace the NRLS and address these limitations [44].

The reasons for submitting reports are also complex, meaning there will be a degree of selection bias that it is impossible to quantify [45]. Several reports were excluded (57%) as they contained insufficient detail or were irrelevant to the subject of diagnostic error. Only a limited number of reports could be evaluated for harm severity. There is a risk of detection bias in the selection and subsequent coding of reports, as this depends on the application of the Primary Care Patient Safety (PISA) taxonomy by report raters. We attempted to counteract this with 10% of the reports double-coded, showing a kappa score of 0.868. Scores higher than 0.700 have been accepted in similar research studies [34, 46] and our methods and training have mirrored these previous research studies.

Though we were able to ascertain the frequency of the types of diagnoses mentioned in reports, we do not know what are the commonest conditions that present to emergency departments. It is difficult to determine whether diagnostic errors are reported with the conditions frequently mentioned because these conditions are more prone to diagnostic error or because these conditions are common presentations in the acute care setting.

The number of reports and their breadth across the UK is informative and potentially transferable for looking at common diagnostic errors nationally. Consistent patterns and inferences, particularly for important conditions or contributory factors, enable the identification of interventions that could be applied to all emergency departments. We could find no previous studies of this size that have analysed patient safety incident reports of diagnostic error occurring in emergency departments.

### Comparison with the literature

The high levels of insufficient assessment reports across a number of diagnoses suggest that there are common sources of these types of errors. These include cognitive and system errors [47]. Cognitive errors are recognised in most cases [17], and are often related to clinician expertise and experience [48]. These human mistakes can be worsened in the emergency department by time constraints on staff for patient assessment and investigation [49].

Several interventions have been suggested to reduce the occurrence of diagnostic error cases. Few of these suggestions have been tested in clinical trials [50, 51]. System-based modifications that optimise clinician skills and use processes for mitigating errors have been shown to reduce the rates of adverse events significantly [52]. Simple programmes, including a whole systems examination intervention [53], assigned training in electrocardiograms (ECG) interpretation [54] and diagnostic checklists [50] can be effective in localised settings. Alongside formal emergency department staff teamwork training [55], these could help mitigate contributory factors, such as limitations in knowledge and cognitive mistakes, and reduce rates of diagnostic error.

Imaging errors, encompassing failure to image appropriately and errors in interpretation, featured prominently in our analysis. Measures that support junior colleagues to more accurately interpret investigations could reduce the number of diagnostic errors [56]. Potential strategies have been cited [57], with recognition that interventions should focus on adapting both educational and system approaches. Changes in both these elements are needed to successfully reduce diagnostic investigation interpretation errors [58]. Radiology interventions can be non-technological, such as structured reporting [59] or double reading [60] of imaging results, or technological, such as perceptual feedback or attentional guidance [61]. Though these interventions show promise, it is unlikely the majority of emergency department clinicians will reach the same technical standard as radiologists [62]. Thus more prompt secondary reviews of radiographs are also needed to reduce the impact of missed fractures [63]. Few of these interventions have been tested [64] but some have shown promise including radiological checklists [64] and computer-aided detection [65].

Other identified errors in our analysis included failure to correctly interpret and follow up other investigations including laboratory results and ECGs. Both continuous education feedback strategies [66] and standardised forms to drive follow-up of investigations are effective interventions [67]. Specific diagnoses, such as abdominal aortic aneurysms, require specific interventions that address challenges in their diagnostic pathway. For example, a low threshold for immediate CT scanning and greater involvement of emergency department clinicians in ultrasound examinations may help reduce missed abdominal aortic aneurysms cases [68, 69]. Similar thresholds or decision tools are applicable to detection of high-risk fractures such as hip and cervical spine fractures. Increasing the utility of these tools and awareness of them could improve emergency department diagnosis for these patients [70, 71].

Diagnostic error is a challenging field to act upon [72–74] but opportunities for improvement can be addressed using a Plan-Do-Study-Act model and through system quality improvement [75]. Small adaptations, across the drivers of Fig. 2, that add up to an overall system modification could help address the multiple causes of diagnostic error and improve emergency department diagnosis. “Blame and shame” approaches do not contribute to learning and system improvement [76]. Instead, future research should be directed towards implementing suggested interventions with a system-oriented direction. These are needed alongside cultural shifts and organisational restructure to be sustainable [77].

## Conclusion

Our study demonstrates that there are multiple opportunities to reduce diagnostic error in the emergency department. Clinicians must have better support in performing patient assessment and interpreting investigations. Interventions to reduce diagnostic error in the emergency department setting could include standardised checklists, structured reporting and technological investigation improvements.

## Data Availability

No further data are available for review.

## Abbreviations

CT: Computed Tomography
DF: Dynamic flexion/extension X-ray studies with Fluoroscopy
DPSIMS: Development of the Patient Safety Incident Management System
ECG: Electrocardiogram
MDCT: Multidetector Computed Tomographic Angiography
MDCTA: Multidetector Computed Tomographic Angiography
MRI: Magnetic Resonance Imaging
NEXUS: National Emergency X-Radiography Utilization Study
NHS: National Health Service
NRLS: National Reporting and Learning System
PISA: Primary Care Patient Safety
RCT: Randomised Control Trial
UK: United Kingdom
USA: United States of America
WHO: World Health Organisation
